# An ageing biomarker signature predicts chronic disease cluster trajectories, physical function and mortality: validation in the TILDA and HRS cohorts

**DOI:** 10.1093/ageing/afag135

**Published:** 2026-05-20

**Authors:** Belinda Hernández, Eric Klopack, Frank Moriarty, Padraic Fallon, Nollaig Bourke, Eileen Crimmins, Rose-Anne Kenny, Cathal Mc Crory, Aisling O'Halloran

**Affiliations:** The National Center for Pharmacoeconomics (NCPE), St James’s Hospital, Dublin, DO8 XN61, Ireland; The Irish Longitudinal Study on Ageing (TILDA), Trinity College Dublin, Dublin, D02 R590, Ireland; Davis School of Gerontology, University of Southern California, Los Angeles, CA 90089, USA; HRB Centre for Primary Care Research, RCSI University of Medicine and Health Sciences, Dublin, D02 YN77, Ireland; Trinity College Dublin, Dublin D02 PN40, Leinster, Ireland; Trinity College Dublin, Dublin D02 PN40, Leinster, Ireland; University of Southern California, Los Angeles, CA 90089, USA; Medical Gerontology, University of Dublin Trinity College, Dublin, D08 NHY1, Ireland; Mercer’s Institute for Successful Ageing (MISA), St James' Hospital, Dublin, D08 E9P6, Ireland; Discipline of Medical Gerontology, Trinity College Dublin, Dublin, D08 XN61, Ireland; Medical Gerontology, Trinity College Dublin, Dublin, D08 XN61, Ireland

**Keywords:** ageing biomarker signature, multimorbidity trajectories, disease clusters, older people

## Abstract

**Background:**

Global population ageing is progressing at an unprecedented rate. Early identification of subpopulations at risk of chronic diseases could mitigate deteriorating health and increasing health service use, while revealing key biological cues and therapeutic targets. We hypothesised that a multisystem biomarker signature could identify future chronic disease trajectories, multimorbidity, functional decline and mortality.

**Methods:**

Eighteen blood biomarkers, representing key systems that become dysregulated with ageing, were assessed among *n* = 4961 participants aged 50+ years from Ireland. Probabilistic clustering classified participants as belonging to one of three biomarker signatures at baseline. Biomarker signatures were designated as low, medium and high risk based on relative levels of biomarker dysregulation within the signatures and previously described associations with chronic disease and mortality. These biomarker signatures were utilised to predict 4-year functional decline; 8-year cardiovascular disease (CVD), diabetes, frailty, disability and 12-year mortality. Results were validated in a US cohort (*n* = 3914).

**Results:**

Low (58.5%), medium (9.2%) and high-risk (32.3%) biomarker signatures were identified in the cohort from Ireland. The high-risk signature was associated with higher 12-year mortality (HR: 1.89, *P* < .001); higher 8-year incidence of CVD, diabetes, multimorbidity, frailty and disability (OR range: 1.46–2.49. *P* < .05); and lower 4-year physical function (*P* < .01). Findings were corroborated in the US cohort. We identified and tracked 6 disease classes over 8 years: healthy, arthritis, diabetes/angina, hypothyroid/osteoporosis/respiratory, vision/anxiety/CVD and multisystem. Associations between the high-risk biomarker signature and two of the five 8-year incident disease classes were observed, implicating dysregulated immune and cardiometabolic pathways.

**Conclusions:**

This study provides evidence that biomarker signatures and profiling of disease patterns can be used to risk stratify and identify ageing subpopulations that would benefit most from targeted preventative or secondary intervention strategies.

Key points

- Developed a novel biomarker signature using an Irish dataset which predicted of 12-year mortality; 8-year incident CVD, diabetes, multimorbidity, frailty and disability.
- Results were validated and corroborated on a separate US cohort.
- Can be used for risk stratification and targeting for preventative or secondary intervention strategies.

## Introduction

Global population ageing has reached unprecedented levels, yet the gap between lifespan and healthspan—the years lived free of disease and disability—remains substantial and may be increasing. A study of 183 countries estimated a mean healthspan-lifespan gap of 9.6 years, with women experiencing a 2.4-year larger gap due to a higher burden of noncommunicable chronic conditions [1]. Up to one-fifth of life may now be lived with chronic disease [2, 3]. Chronic conditions in mid- and later life account for four out of five years lived with disability, while cardiovascular disease, cancer, diabetes and respiratory disease make up 80% of chronic disease deaths [4, 5]. Despite differing clinically, these diseases share risk across the life-course, suggesting common biological signatures that could help predict who is at risk for deteriorating health.

Multimorbidity—living with multiple chronic conditions—is a growing challenge. It affects 37% of European Union (EU) adults aged ≥65 years [6] and 81% of older adults in the US [7]. The implications for individuals, governments and health systems are profound [8–12]. Consequently, research into multimorbidity patterns has expanded rapidly [6], with our group and others identifying chronic disease profiles across ageing populations globally [13–18]. However, most studies are cross-sectional and fewer explore longitudinal trajectories and transitions [6, 17, 19]. Yet people sharing disease patterns often share risk factors, healthcare needs and outcomes. Longitudinal characterisation is therefore essential for identifying those at risk of worsening health and aligns with Priority 1 of the Academy of Medical Sciences global priorities (2018).

Ageing is dynamic and heterogeneous, with marked variation in biological ageing rates [20]. Biological ageing arises from accumulating molecular, cellular, physiological and functional dysregulation, leading to chronic disease and progressive outcomes along the ‘disability cascade,’ including functional decline, frailty, disability and death [21, 22]. Divergence in ageing trajectories becomes more pronounced at older ages [22, 23]. Evidence suggests shared causal mechanisms underlie clusters of chronic disease, and the onset of one chronic condition accelerates others [24]. Geroscience research highlights dysregulated biological pathways as common mechanisms driving clusters of chronic disease and outcomes on the disability cascade [25–27].

To examine how biological pathways change with age, Ahadi and colleagues performed deep longitudinal ‘omics’ profiling in 43 adults aged 27–75 years, identifying 4 biological ‘ageotypes’ representing distinct ageing pathways [28]. Tian and colleagues modelled biological ageing across 10 organ systems using blood, physiological and neuroimaging phenotypes from 143 423 healthy UK Biobank participants, revealing heterogeneous organ-specific ageing patterns predictive of chronic disease and mortality [29].

Despite these advances, studies linking biological ageing patterns captured through blood-based and physiological phenotype, to long-term chronic disease trajectories remain limited. Here, we investigate circulating blood biomarker data across a range of biological systems and common age-related chronic conditions in The Irish Longitudinal Study on Ageing (TILDA) to identify stable biomarker signatures associated with profiles of chronic disease and disability cascade outcomes (functional decline: low grip strength, slow gait speed and timed up and go; frailty, disability and mortality). We also demonstrate the broader cross-national and cross-cultural implications of our findings by validating them using harmonised data from the Health and Retirement Study (HRS) in the US.

## Methods

### TILDA cohort sample

Data were obtained from individuals who participated in waves 1–5 (2009–2018) of TILDA, a nationally representative study of community-dwelling adults aged 50+ in Ireland [30–32]. Ethical approval for the study was provided by the Faculty of Health Science Research Ethics board in Trinity College Dublin. Informed consent was obtained from all respondents during data collection adhering to the Declaration of Helsinki. The analytical sample of 4961 participants and sample inclusion criteria are provided in Appendix S1 of the Supplementary Data section.

### HRS validation cohort sample

The HRS is a nationally representative sample of Americans over the age of 50. TILDA is modelled on the HRS; its data are harmonised to the HRS and follows the same data collection protocols. The HRS was approved by the University of Michigan Health Sciences/Behavioural Sciences Institutional Review Board. Data were available for a subsample of individuals (*N* = 4104) who participated in the HRS 2016 Venous Blood Study and had the blood biomarker assays completed at the University of Minnesota Advanced Research and Diagnostic Laboratory as described previously [33]. Participants were excluded from the present analysis if they were not age eligible for HRS or had missing data on the outcomes or covariates. The final HRS validation sample with data for 18 blood biomarkers, health outcomes and covariates included 3914 participants.

### Statistical methods

#### Biomarker correlations and signatures

A correlation network between the selected biomarkers was generated from a Pearson pairwise correlation matrix.

**Figure 1:**
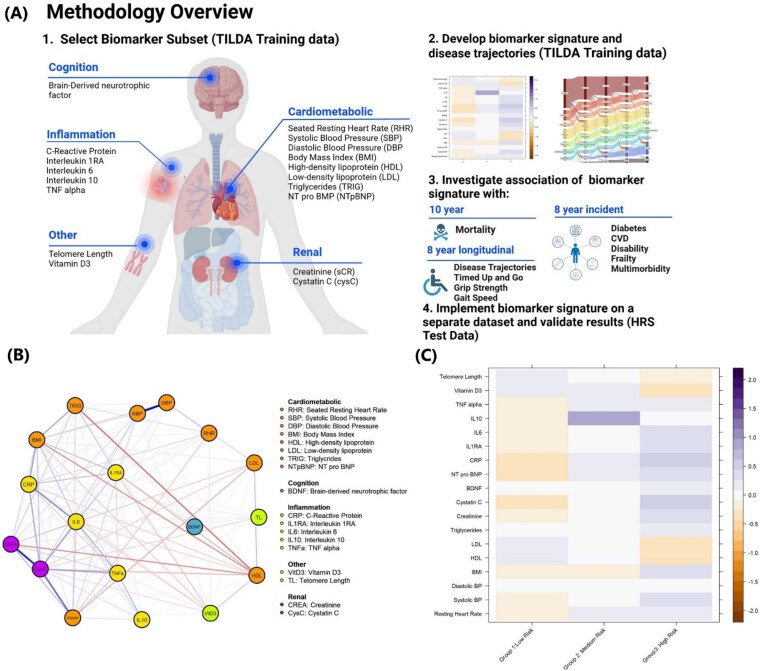
Identification of a biomarker signature. (A) Overview of the study design. (B) Correlation network of the selected biomarkers. Red lines connecting nodes indicate negative correlation and blue a positive correlation. Line thickness and colour intensity is proportional to the strength of the correlation. (C) Heatmap of the mean value/profile for the 18 individual biomarkers across each of the 3 biological clusters. Note all biomarkers were significantly different between clusters even after correction for multiple testing. Group 1: low risk multisystem homeostasis (stable with reduced cardiac, renal and pro-inflammatory biomarker risk profile). Group 2: medium risk: early multisystem dysregulation (slightly elevated heart rate, pro-inflammatory, neurocognitive biomarker risk profile). Group 3: high risk: multisystem dysregulation (high cardio-metabolic, pro-inflammatory, renal and neurocognitive biomarker risk profile).

Plasma biomarker signatures were developed based on 18 biomarkers across multiple biological systems using the TILDA data and implementing model-based clustering (Figure 1A and Appendices S2 and S3 in the Supplementary Data section). These 18 biomarkers were selected based on their known associations with a wide array of age-related chronic conditions and also their availability for validation in the HRS data set.

#### Confounders

The following variables were controlled for in multivariate models on TILDA data unless otherwise stated: age, sex, education, smoking history (former, current, never), use of lipid-modifying drugs identified as uniquely prescribed for those with high cholesterol [i.e. use of medications with WHO Anatomical Therapeutic Chemical (ATC) code C10 (excluding C10AX06)] and use of anti-hypertensives uniquely prescribed for hypertension [identified through use of beta-blockers (C07AS, C07AG), calcium channel blockers (C08C, C08D, C08E, C08G), thiazide diuretics (C03A), alpha-adrenoceptor antagonists (C02CA, C02LE), angiotensin-converting enzyme inhibitors (C09A, C09B) or angiotensin-II receptor blockers (C90C, C09D)]. Due to data availability only age, race, sex, education and smoking were controlled for in HRS validation models. Race was not controlled for in models trained on TILDA data as the sample consisted exclusively of white Irish participants.

#### Twelve-year mortality

A Cox proportional hazards model tested the association between baseline biomarker signatures and 12-year mortality, adjusting for covariates and confounders previously described. Schoenfeld residuals were used to assess the validity of the proportionality assumption. There was no evidence of time dependence among the covariates.

#### Eight-year incident disease

Logistic regression was used to model associations between the biomarker signatures and 8-year incident outcomes (CVD, diabetes, multimorbidity, frailty, disability and incident disease clusters). Binary incident disease variables were created for presence/absence of each condition over 8 years among those who were disease free at baseline. All models were fully adjusted as previously described.

#### Functional decline

TUG time, grip strength and gait speed were used to assess functional decline. TUG time was assessed longitudinally over 8-year follow-up. Grip strength and gait speed were assessed longitudinally over a 4-year follow-up due to data availability. These outcomes were modelled using mixed effects linear models adjusting for the confounders previously described and an additional covariate denoting the year of follow-up. An interaction term between biomarker signature and year of follow-up was also included when significant.

#### Disease trajectories

To identify underlying groups of diseases over an 8-year period, a latent Markov model with a multivariate categorical response was implemented (Appendix S2 in the Supplementary Data section).

## Results

### Development of multisystem biomarker signatures

A high positive intercorrelation was observed among the inflammatory markers, markers of renal function, blood pressure measures and between BMI and inflammatory markers (Figure 1B).

Three main biomarker signatures were identified from the unsupervised clustering of 4961 participants (Figure 1C and Appendix S4  Supplementary Figure S2 in the Supplementary Data section). Biomarker signatures were designated as low, medium and high-risk based on relative levels of biomarker dysregulation within the signatures and previously described associations with chronic disease and mortality (see Appendix S3 in the Supplementary Data section). All markers differed significantly between signatures (*P* < .001) even after correction for multiple testing. The prevalence of each biomarker signature at baseline was low (*n* = 2902, 58.5%), medium (*n* = 456, 9.2%) and high-risk (*n* = 1603, 32.3%). Compared to the low-risk biomarker signature, the medium and high-risk signatures had progressively higher CRP, IL-6, IL1-RA, cystatin C, creatinine, NT-pBNP and SBP but progressively lower LDL, HDL and leukocyte telomere length. Within the high-risk cluster, vitamin D3 was lowest, while BMI, triglycerides and BDNF were highest. The medium and low-risk signatures had similar levels of these biomarkers. TNF-α and RHR were higher but similar in the medium and high-risk groups. DBP varied least across groups. Of note was IL-10, which was highest in the medium-risk group, lowest in the low-risk group and at an intermediate level in the high-risk group (Figure 1C).

Cross-sectionally, increasing age, adverse smoking history, more multimorbidity, higher use of lipid-lowering and anti-hypertensive medications, lower educational attainment and lower physical activity were progressively associated with the medium and high-risk biomarker signatures (Table 1).

**Table 1:** Breakdown of participant characteristics by biomarker signature.

	Group 1: low risk (58.5%, *n* = 2902)	Group 2: medium risk (9.2%, *n* = 456)	Group 3: high risk (32.3%, *n* = 1603)	*P*-value
Age (mean, sd)	60.1 (7.39)	62.8 (9.18)	67.1 (9.92)	<.001
Education (*n*, %)				<.001
Primary	573 (19.75)	108 (23.68)	585 (36.49)	–
Secondary	1223 (42.14)	192 (42.11)	619 (38.62)	–
Third level	1106 (38.11)	156 (34.21)	399 (24.89)	–
Sex (*n*, %)				.028
Male	1347 (46.42)	189 (41.45)	777 (48.47)	–
Female	1555 (53.58)	267 (58.55)	826 (51.53)	–
Multimorbidity (*n*, %)				<.001
0	1121 (38.63)	113 (24.78)	316 (19.71)	–
1	846 (29.15)	130 (28.51)	394 (25.58)	–
2+	935 (32.22)	213 (46.71)	893 (55.71)	–
Physical activity (*n*, %)				<.001
Low	700 (24.30)	128 (28.57)	618 (38.82)	–
Medium	1048 (36.38)	158 (35.27)	527 (33.10)	–
Vigorous	1133 (39.33)	162 (36.16)	447 (28.08)	–
Anti-hypertensive Medication use (*n*, %)	702 (24.19)	158 (34.65)	897 (55.96)	<.001
Lipid-modifying medication use (*n*, %)	729 (25.12)	120 (26.32)	692 (43.20)	<.001
Smoking (*n*, %)				<.001
Never	1430 (49.28)	200 (43.86)	612 (38.18)	–
Past	1089 (37.53)	188 (41.23)	653 (40.74)	–
Current	383 (13.20)	68 (14.91)	338 (21.09)	–

### Biomarker signature and risk for age-related health outcomes

**Figure 2:**
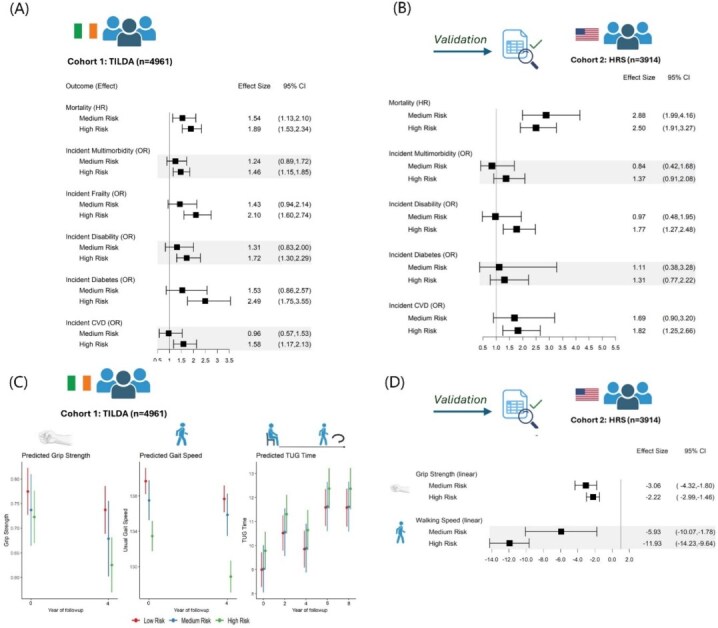
Biomarker signature identifies groups at risk for worsening health outcomes across cohorts. (A) Forest plot showing associations between the 3 biomarker signatures on 12-year time to mortality and 8-year incident disease and 4-year functional outcomes for the TILDA cohort. Low risk is the reference class. (B) Forest plot showing associations between the 3 biomarker signatures, 4-year mortality and 4-year incident outcomes for HRS validation dataset. Low risk is the reference class. (C) Adjusted predicted grip strength, gait speed and TUG time by biomarker signature for TILDA cohort. (D) Forest plot showing associations between the 3 biomarker signatures and walking speed and grip strength in the HRS validation dataset. Low risk is the reference class.

We next aimed to determine whether these biomarker signature clusters could predict subsequent health outcomes. When compared to our identified low-risk group, both medium and high-risk biomarker signatures had progressively increased hazard of 12-year mortality (HR: 1.54, 95% CI: 1.13–2.10) and (HR: 1.89, 1.53–2.34), respectively (Figure 2A). The high-risk group also had significantly higher odds of 8-year incident: cardiovascular disease (OR: 1.67, 95% CI: 1.22–2.30), diabetes (OR: 2.49, 95% CI: 1.75–3.55), multimorbidity (OR: 1.46, 95% CI: 1.17–1.83), frailty (OR: 2.1, 95% CI: 1.60–2.74) and disability (OR: 1.72, 95% CI: 1.30–2.29), after adjustment for confounders. The biomarker signatures also had a dose response relationship with functional status (Figure 2C) with the high-risk group exhibiting the lowest grip strength, slowest walking speed and slowest TUG time at baseline and follow-up. However, there was no evidence of an interaction effect between the biomarker signatures and TUG time at follow-up.

To confirm our findings, we applied this clustering approach to an independent cohort of community-dwelling older adults in the US, the HRS cohort (*n* = 3914). Associations between the biomarker signatures and 4-year mortality and incident health outcomes were tested in the HRS validation dataset (Figure 2B; *n* = 3914 mortality; *n* = 1779 incident disability, *n* = 1072 incident multimorbidity; *n* = 2140 incident CVD; *n* = 2209 incident diabetes). The medium and high-risk biomarker signatures were associated with higher mortality (HR: 2.88, 95% CI: 1.99–4.16) and (HR: 2.50, 95% CI: 1.91–3.27), respectively. The concordance for mortality prediction was high for both training and test datasets (0.82 for TILDA and 0.79 for HRS). Furthermore, the high-risk biomarker signature was significantly associated with 4-year incident disability (OR: 1.77, 95% CI: 1.27–2.48) and incident CVD (OR: 1.82, 95% CI: 1.25–2.66) after adjustment for age, sex, education and smoking history for the HRS cohort. In cross-sectional analysis of physical function in the HRS cohort, the medium (Coeff: −3.06, 95% CI: −4.32, −1.80) and high-risk (Coeff: −2.22, 95% CI: −2.99, −1.46) biomarker signatures were associated with lower grip strength (Figure 2D). Slower walking speed was also associated with the medium (Coeff: −5.93, 95% CI: −10.1, −1.8) and high-risk (Coeff: −11.9, 95% CI: −14.2, −9.6) signatures.

### Disease classes and trajectories

Six disease trajectories were identified over an 8-year follow up period (see Appendix S4  Supplementary Figure S2 in the Supplementary Data section). Their average prevalences were: healthy (average prevalence over 8-years 27.1%); arthritis (19.7%); multisystem (17.4%); hypothyroid/osteoporosis/respiratory (16.1%); vision/anxiety/CVD (10.9%); and diabetes/angina (8.8%).

**Figure 3:**
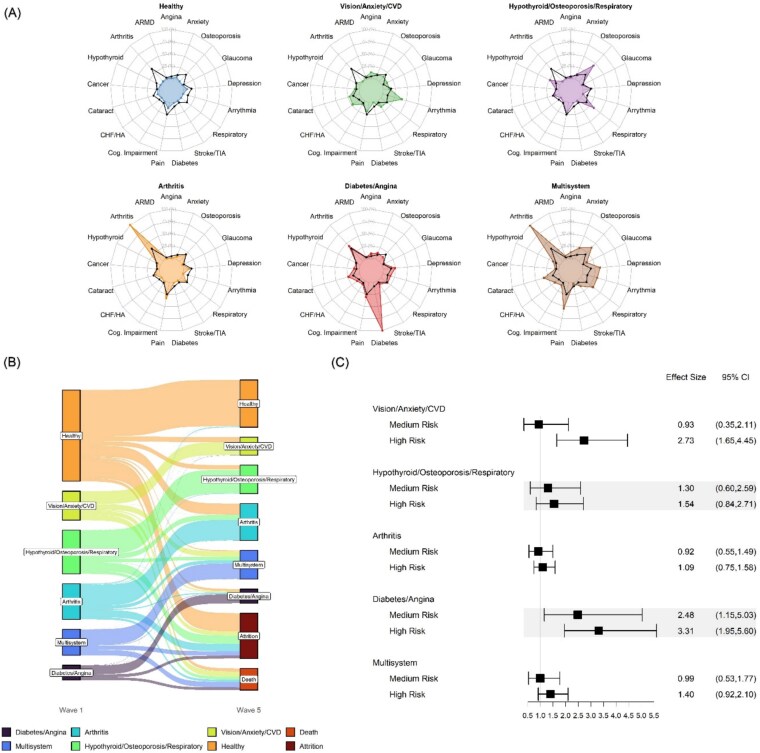
Biomarker signatures associate with 8-year disease cluster trajectories. (A) Disease profiles. Black dots denote overall population prevalence of a given condition. Filled/coloured areas denote the prevalence of the disease within a given cluster. (B) Alluvial plot of migrations between disease profiles from baseline to 8-year follow-up. A more detailed version of this diagram showing individual transitions across all 5 data collection timepoints (baseline, 2, 4, 6 and 8-year follow up) can be seen in Supplementary Figure S3. (C) Forest plot showing the coefficient for baseline biomarker signatures on 8-year incident disease profiles after adjustment for age, sex, education, smoking history, lipid-lowering and anti-hypertensive medications. Low risk biomarker signature is the reference.

The transitions between disease classes over time are presented in Figure 3B (see also Appendix S4  Supplementary Figure S3 which shows transitions by each follow-up year). By 8-year follow-up, from within the healthy class at baseline: 26% transitioned to a higher disease class, 20% were lost to attrition and 3% were deceased. Notably, the number of participants belonging to the healthy class almost halved, dropping from 1674 to 848 participants as they transitioned to higher risk disease classes. Mortality was also lowest in this class.

There was moderate stability for three of the disease classes over time with 60% of diabetes/angina, 60% of multisystem and 58% of arthritis at baseline remaining in the same disease class over 8 years. Furthermore 54% of the hypothyroid/osteoporosis/respiratory and 44% of the vision/anxiety/CVD classes at baseline remained in the same disease class 8 years later. Approximately 19% of people who started in the least stable vision/anxiety/CVD class at baseline moved to the multisystem class, 4% transitioned to the diabetes/angina class, 15% were lost to attrition and 18% were deceased. The largest transitions from the hypothyroid/osteoporosis/respiratory class at baseline were to the arthritis class (12%), followed by the multisystem class (8%), with 19% lost to attrition and 7% were deceased by 8 year follow-up; 2% of individuals in the multisystem class at baseline transitioned to the diabetes/angina class, 17% were lost to attrition and 21% were deceased by 8 year follow-up. Of those belonging to the arthritis class at baseline, 9% transitioned to the multisystem class, 3% transitioned to the diabetes/angina class, 24% were lost to attrition and 6% were deceased by follow-up.

### Biomarker signature at baseline and 8-year incident disease classes

The baseline biomarker signatures predicted higher odds of two of the five disease classes compared to the low-risk class (Figure 3C). The high-risk biomarker signature was significantly associated with higher odds of 8-year incident vision/anxiety/CVD (OR: 2.73, 95% CI: 1.65–4.45). Both the medium (OR: 2.48, 95% CI 1.15, 5.03) and high (OR: 3.31, 95% CI: 1.95–5.60) risk signatures were associated with 8-year incident diabetes/angina class. No association was observed between the biomarker signature at baseline and the hypothyroid/osteoporosis/respiratory, arthritis nor the multisystem disease classes.

### Disease classes and mortality risk

The vision/anxiety/CVD class had the highest risk of mortality (HR: 2.10, 95% CI: 1.51–2.92) followed by the multisystem (HR: 1.89, 95% CI: 1.34–2.67), hypothyroid/osteoporosis/respiratory (HR: 1.65, 95% CI: 1.16–2.37), arthritis (HR: 1.58, 95% CI: 1.11–2.25) and angina/diabetes (HR: 1.58, 95% CI: 1.11–2.25) class. Participants in the vision/anxiety/CVD class at baseline accounted for 25.7% of all mortalities that had occurred at 8-year follow-up, followed by the multisystem (23.4%), diabetes/angina (18.1%), arthritis (9.51%) and hypothyroid/osteoporosis/respiratory (7.88%) class (Table 2).

**Table 2:** Results from Cox-PH model showing the association between baseline disease states and 12-year mortality after adjustment for age, sex, education, smoking history and use of antihypertensive and lipid modifying medications at baseline.

Baseline disease state	HR (95% CI)	*P*-value	Baseline Disease State n (%)	Deceased by 12 years n (%)
Healthy	REF	REF	1674 (38.0)	67 (4.0)
Diabetes/angina	1.57 (1.05, 2.36)	.029	271 (6.2)	49 (18.1)
Arthritis	1.58 (1.11, 2.25)	.012	652 (14.8)	62 (9.5)
Hypothyroid/osteoporosis/respiratory	1.65 (1.16, 2.37)	.006	800 (18.2)	63 (7.9)
Multisystem	1.89 (1.34, 2.67)	<.001	476 (10.8)	111 (23.3)
Vision/anxiety/CVD	2.10 (1.51, 2.92)	<.001	530 (12.0)	122 (23.0)

## Discussion

In this study, we demonstrate that common blood-based biomarkers can be combined into biologically coherent signatures that consistently predict long-term health outcomes across two large, independent ageing cohorts. Most notably, we show that the high-risk biomarker signature is strongly linked not only to outcomes on the disability cascade (functional decline, frailty, disability, mortality), but also chronic disease and transitions into specific multimorbidity clusters over 8 years. These findings provide novel insight into how early multisystem dysregulation contributes to the emergence and progression of complex disease patterns in later life.

The biomarker signatures were constructed from 18 routinely measured indicators reflecting immune, cardiometabolic, renal, musculoskeletal, cognitive and senescence-related processes known to deteriorate with age [21, 22]. Probabilistic clustering identified three signatures designated as low-, medium- and high-risk, representing increasing levels of multisystem physiological dysregulation. This interpretation is reinforced by the clear dose–response gradient observed across leukocyte telomere length, with the shortest telomeres in the high-risk group, consistent with previous evidence linking telomere attrition to accelerated biological ageing and chronic disease including CVD [26, 34].

One of the most compelling findings is the association between the high-risk biomarker signature and progression into the diabetes/angina and vision/anxiety/CVD disease classes over 8 years. These clusters were characterised by high burdens of cardiometabolic dysfunction, inflammatory conditions and CVD. The enrichment of inflammatory and cardiometabolic biomarkers, higher CRP, IL-6, IL-1RA, NTproBNP, SBP, creatinine and cystatin C, alongside lower vitamin D3, HDL, LDL and IL-1, in the high-risk group is biologically plausible given the aetiology of these disease classes. Inflammatory cytokines and impaired lipid metabolism play central roles in diabetes and atherosclerotic disease [35–39], while NT-proBNP reflects cardiac stress and failure. Elevated creatinine and cystatin C signal early renal decline and have well-established associations with type 2 diabetes and adverse CVD outcomes [40–42]. Likewise, systemic inflammation and low vitamin D levels have been implicated in hypothyroidism, osteoporosis and anxiety disorders [43–47]. Increased CRP, IL-6, cystatin C, NT-proBNP and decreased vitamin D and telomere length have also been shown to predict health outcomes and mortality in older adults [48, 49].

The pattern of IL-10 across biomarker signatures is particularly interesting. IL-10 was higher in the medium-risk group but relatively lower in the high-risk group, despite higher pro-inflammatory cytokines in the latter. IL-10 is a pleiotropic cytokine with context-dependent anti- and pro-inflammatory actions; it increases in response to IL-6 and modulates TNF-α [50, 51]. The medium-risk elevations may reflect a hormetic, compensatory response, whereby low-level inflammatory cytokine signalling induces IL-10, while chronically high inflammatory load in the high-risk group may inhibit IL-10 expression.

The relationship between biomarker signatures and disease class trajectories adds a dynamic dimension to our findings. Participants in low-burden classes often transitioned into more complex multisystem classes over 8 years, and these complex classes had the highest mortality risk, largely due to their cardiovascular burden [52]. Importantly, those entering high-risk disease classes tended to originate from high-risk biomarker groups, supporting the role of early multisystem dysregulation as a driver of multimorbidity progression. Individuals in intermediate classes may therefore represent key targets for preventative interventions.

Beyond disease clustering, the biomarker signatures were strongly associated with conventional clinical outcomes. After adjustment for demographic, lifestyle and health factors, the high-risk signature predicted incident CVD, type 2 diabetes, multimorbidity, functional decline, frailty, disability and mortality over follow-up periods ranging from 4 to 12 years. The medium-risk signature showed similar directional trends but reached significance only for physical function measures and mortality; this may reflect reduced statistical power due to smaller sample size or that this group occupies an intermediate state where intervention could be most effective. Meanwhile, the low-risk signature was consistently associated with the best outcomes, supporting the concept that maintaining multisystem homeostasis is protective in later life [21, 22, 53].

The physical function outcome of gait speed, grip strength and Timed Up and Go showed strong associations with the high-risk signature, mirroring incident disease patterns. These metrics are established indices of frailty, disability and mortality [20, 28, 54, 55]. Their strong link with the high-risk signature likely reflects their sensitivity to multisystem physiological decline.

Our work builds on previous studies applying deep phenotyping or biomarker-disease clustering approaches [28, 29, 56] and research examining multimorbidity trajectories over time [5, 16, 18]. The study by Murad and Melamud (2022) identified disease clusters using nonlinear dimensionality reduction of 41 biochemical and CBC parameters in the UK Biobank, demonstrating the predictive capacity of biomarker patterns [57]. However, our study differs in key ways: biomarker signatures and disease classes were derived independently, and we examined disease cluster transitions longitudinally. Having three signatures and six disease classes also enhances clinical interpretability and potential utility.

A major strength of the study is its validation in an independent cohort. Using harmonised data from the US Health and Retirement Study, we replicated associations between the high-risk signature and physical function, incident CVD, disability and mortality. Despite being trained in a genetically and culturally homogenous Irish sample, the signature performed robustly in a more racially diverse US cohort. These cross-cohort findings support the argument that the relationships identified reflect fundamental biological ageing processes rather than cohort-specific artefacts. Other strengths include the large, well-characterised TILDA cohort; clinically accessible biomarkers; integration of functional, multimorbidity and mortality outcomes; and the capacity to track disease class transitions over time.

Limitations include biomarker selection constrained by availability in both cohorts, potentially limiting the representation of all ageing systems. Biomarkers were measured at a single baseline time point; although many cytokines show longitudinal stability and are suitable for epidemiological studies [51], repeated measures would enhance causal inference. Epigenomic surrogates of biomarkers may offer more stable alternatives where available [58]. Adjusting for medications to treat hypertension and hypercholesterolemia may have resulted in model overfit, however, we have repeated the analysis without these confounders, and there was no material change to the results presented. Cause-specific mortality analyses were not possible due to limited power, and unmeasured confounding cannot be excluded.

Despite these limitations, our findings provide strong evidence that routinely measured biomarkers can be combined into meaningful signatures reflecting multisystem ageing and predicting complex disease trajectories. Identifying individuals at high risk of transitioning into cardiometabolic–inflammatory disease classes has clear potential for targeted intervention and improved preventative strategies in ageing populations.

## Conclusions

In conclusion, we show that routinely measured biomarkers can be combined into robust signatures that reflect multisystem biological ageing and predict long-term functional decline, multimorbidity trajectories and mortality. The high-risk signature identified individuals most likely to transition into complex cardiometabolic and inflammatory disease classes, highlighting its potential utility for early risk stratification. Validation in an independent cohort underscores the generalisability of these findings. Although biomarker availability and single baseline measurements impose limitations, this work demonstrates the value of cohort studies of ageing with accessible clinical biomarkers for identifying vulnerable subgroups and informing targeted prevention strategies in ageing populations.

## Supplementary Material

afag135_Supplementary_materials

## Data Availability

The dataset(s) supporting the conclusions of this article are not publicly available due to data protection regulations but are accessible at TILDA on reasonable request. The procedures to gain access to TILDA data are specified at https://tilda.tcd.ie/data/accessing-data/. Data code can be found in Appendix 5 of the Supplementary Data section.
