# Supplementary material for: An ageing biomarker signature predicts chronic disease cluster trajectories, physical function and mortality: validation in the TILDA and HRS cohorts
Source: Age Ageing. 2026 May 20;55(5):afag135. doi: 10.1093/ageing/afag135 (PMC13189271; doi:10.1093/ageing/afag135)
Supplement: afag135_Supplementary_materials [file afag135_supplementary_materials.docx]

# An ageing biomarker signature predicts chronic disease cluster trajectories, physical function and mortality: Validation in the TILDA and HRS cohorts.

**Appendix 1.** Sample inclusion criteria for initial analysis of biomarker clusters in the TILDA cohort. Page 2

**Appendix 2** Definitions of chronic conditions in the TILDA and HRS cohorts.

with supplementary references. Page 3

**Appendix 3** Summary of function, body system and associated outcomes for 18 biomarkers measured in TILDA and HRS cohorts. Page 7

**Appendix 4**

**Supplementary Figure 1.** Integrated Complete-data Likelihood (ICL) criterion for 1 to 9 clusters over 14 different constraints on the eigenvalue decomposition of the covariance matrix for TILDA biomarker data. The three cluster solution using the VVE distribution structure which assumes an ellipsoidal distribution with variable volume and equal shape and orientation were chosen as this was the solution which maximised the ICL. Page 10

**Supplementary Figure 2.** Bayesian Information Criterion (BIC) for 1 to 10 disease classes using TILDA data. Six classes were chosen in the final model as there were only very incremental improvements in the BIC beyond this point. Page 11.

**Supplementary Figure 3**. Alluvial plot showing participant transitions between disease classes at each wave of data collection over 9 years I the TILDA cohort. Page 12.

**Appendix 5** Supplementary Code Page 13.

# Appendix 1

**. Sample inclusion criteria for initial analysis of biomarker clusters in the TILDA cohort.**

|  | **Samples Removed** | **Total Sample** |
| --- | --- | --- |
| Total participants in Wave 1 |  | 8501 |
| Participants aged <50 at baseline | (330) | 8171 |
| Remove participants who did not have blood test performed | (2270) | 5901 |
| Remove participants who did not have full  biomarker panel measured in blood sample | (940) | 4961 |

# Appendix 2

# Supplementary Methods

## Cohort Definitions

## Biomarkers

The biological systems and biomarkers included were as follows. *Neurocognition:* Brain-derived neurotrophic factor (BDNF); *Cardiometabolism:* body mass index (BMI), resting heart rate (RHR), systolic blood pressure (SBP), diastolic blood pressure (DBP), high density lipoprotein cholesterol (HDL), low density lipoprotein cholesterol (LDL), triglycerides (TRIG) and N-terminal pro B-type natriuretic peptide (NTpBNP); *Immune response:* C-reactive protein (CRP), interleukin-1 receptor antagonist (IL-1RA), interleukin-6 (IL-6), interleukin-10 (IL-10), tumour necrosis factor alpha (TNF-α); Musculoskeletal function: vitamin D3 (VitD3); *Renal function:* creatinine (CREA), cystatin C (CYSC); and S*enescence:* leukocyte telomere length (LTL). Height and weight were measured using a SECA 240 wall mounted measuring rod and a SECA electronic floor scales, respectively. BMI was calculated by dividing weight (kg) by height (m) squared. Systolic blood pressure (SBP, mmHg), diastolic blood pressure (DBP, mmHg) and resting heart rate (RHR, bpm) were obtained in the seated position as the mean of two measurements separated by a 1-minute interval using an automatic digital oscillometric blood pressure monitor (OMRONTM, M10-IT). Leukocyte telomere length (LTL) was determined by a monochrome multiplex quantitative PCR (MMQPCR) method as described previously [34]. LTL was calculated as the mean telomere to single copy gene (T/S) ratio and approximates the average telomere length per cell.

A custom V-Plex (IL-6, IL-10, IL-1RA and TNF-α), R-Plex (NTproBNP) and U-plex (BDNF) enzyme-linked immunosorbent assay (ELISA) kit was designed (Meso Scale Diagnostics, MSD, MD, USA). Assays were conducted as per the manufacturers’ instructions with plates read using a MESO QuickPlex SQ 120MM instrument. Protein concentrations (pg/ml) were calculated using Meso Scale Diagnostics Discovery Workbench Software (v4.0). CRP (mg/l), creatinine (µmol/l), cystatin C (mg/l), LDL cholesterol (mmol/l), HDL cholesterol (mmol/l) and triglycerides (mmol/l) were measured using standard clinical laboratory assays using Roche Cobas assay kits and Roche Hitachi Cobas c 701 analyser (Roche Diagnostics GmbH, Mannheim, Germany). Plasma 25-hydroxyvitamin D (25(OH)D) or vitamin D3 (nmol/l) quantification was by LC-MS/MS. Vitamin D analysis included total plasma 25(OH)D (D2 and D3) concentrations which were quantified by a fully validated method (Chromsystems Instruments and Chemicals GmbH; MassChrom 25-OH-Vitamin D3/D2) using liquid chromatography-tandem mass spectrometry (API 4000; AB SCIEX), UK), as previously described [35]. For participants with undetectable levels of analytes in blood plasma, these were replaced by the Lower Limit of Detection (LLOD) for subsequent statistical analysis.

## Development of Biomarker Signatures

Biomarker signatures, or clusters, at baseline wave 1 were identified using model-based clustering, which is a probabilistic or fuzzy clustering algorithm that has the advantage of allowing observations’ data to contribute to multiple clusters as opposed to heuristic-based algorithms such as k-means or hierarchical based clustering. Here we implemented a finite Gaussian mixture model using the R package mclust [41]. One of the main advantages of mixture models in general is that they allow for flexible control over the definition of the shape and structure of the clusters. The volume, shape and orientation of the cluster solution can be assessed by adding various constraints to the covariance matrix; 14 of which are assessed automatically by the mclust software. We varied the number of clusters from 1 to 9 and chose the number of clusters and the covariance structure for our final model based on the Integrated Complete-data Likelihood (ICL) criterion (Supplementary Figure 1). Participants were assigned to the cluster to which they had highest probability of belonging.

Telomere length was assumed missing at random for n=379 participants and cholesterol, LDL, HDL and triglycerides for n=9 participants. Missing data were multiply imputed over 100 independent iterations of the algorithm using the imputeData function in R and the average clustering solution over the 100 independent iterations was obtained to ensure stability of the final solution. A label switching algorithm was employed using the matchLabels function in the Thresher R package to assign observations to signatures over iterations based on majority vote. Univariate mean differences in biomarkers across signatures were assessed using the Kriskal test and p-values corrected for multiple testing using the Bonferroni correction.

## Outcomes

*12-year Mortality:* In Ireland, all deaths are registered through the General Register Office (GRO). Mortality status was determined by inspection of death certificates using data linkage between TILDA and the GRO, the methodology for which has been documented elsewhere [36]. Data are available for all deaths of TILDA participants up to 31st January 2022 (~12 year follow up from baseline). In total there were 584 deaths (11.77%). *Multimorbidity and Medical Conditions:* Multimorbidity for the TILDA data was defined as a binary variable indicating the presence of 2 or more of the following conditions: angina, heart attack, chronic heart failure, heart arrhythmia, diabetes, stroke, transient ischaemic attack (TIA), lung disease, asthma, arthritis, osteoporosis, cancer, cognitive impairment, pain, cataract, glaucoma, age related macular degeneration or depression. Multimorbidity for HRS validation was defined as presence of hypertension, diabetes, cancer, lung disease, heart disease, stroke, and arthritis. Differences in the definition were due to lack of data for the remaining conditions in the HRS data. *Disability:* Disability was defined as a binary variable indicating problems performing ≥1 of the following activities of daily living (ADL) or instrumental activities of daily living (IADL): dressing, walking, bathing or showering, eating, getting in and out of bed or using the toilet, preparing a hot meal, doing household chores, shopping for groceries, making telephone calls, taking medication and managing money. This measure was dichotomised for practicality and due to low sample sizes for many of the individual categories. *Frailty:* Frailty was defined as a binary variable distinguishing non-frail from pre-frail and frail and was operationalised in TILDA based on Fried’s frailty phenotype as described previously [35, 37]. *Grip Strength:* Grip strength during the health assessments at waves 1 and 3 was measured using a hydraulic hand dynamometer (Baseline, Fabrication Enterprises, Inc., White Plains, NY) [38]. The mean value from two measurements using the dominant hand was taken. *Gait Speed:* Gait was assessed using the GAITRite 4.88m computerised walkway during the health assessments at waves 1 and 3. Mean gait speed over two walks at usual pace were used in this study. Participants started and finished their walk 2.5m before and 2m after the end of the walkway to avoid anomalies due to acceleration and deceleration at the start and end of the walk [39]. *Timed Up and Go (TUG):* The TUG test is a simple test used to assess a person's mobility and requires both static and dynamic balance. Participants were asked to stand from a seated position, walk 3 m at their usual pace, turn around, walk back to the chair, and sit down. The chair had armrests, and the seat was 46cm high. TUG was only performed once. Walking aids were allowed. The time taken from the command “Go” to when the participant was sitting with their back resting against the back of the chair was recorded using a stopwatch in seconds (s) and centiseconds (cs) [38, 40].

## Definitions of chronic conditions in TILDA and HRS.

Depression was defined as use of ATC N06A medication and/or a score of 16 or more on the long form Center for Epidemiologic Studies Depression Scale (CES-D) at waves 1 and 2 or a score of 9 or more on the short form CES-D scale, which was performed from wave 3 onwards. The validity, reliability and consistency of these criteria has previously been tested and approved in TILDA [1, 2].

Anxiety was defined as use of the following medications N05B N05CD N05AE N05CF and or a score of 11 or more on the Hospital Anxiety and Depression Scale (HADS) at wave 1 and identified using the Composite International Diagnostic Interview–Short Form (CIDI-SF) from wave 2 onwards.

Cognitive impairment was defined as having a global cognition score of 1 standard deviation or more below the mean. The composite global cognition score was derived by combining scores across tests for verbal memory, executive function and orientation as described in TILDA [3]

The following conditions were identified by a combination of self-reported doctor’s diagnosis and/or use of the medications based on their ATC codes: diabetes (A10A, A10B, A10X), osteoporosis (M05BA, M05BB, M05BX03, M05BX53, M05BX04, H05AA02, H05AA01, H05AA03, H05BA, G03XC01), cancer (L01 L02).

Respiratory illnesses included self-reported asthma, COPD or lung disease and/or use of any of the following medications: R03BA, R03AK06, R03AK07, R03AK08, R03AK09, R03AK10, R03AK11, R03AK12, R03AK13, R03AC02, R03AC03, R03AK04, R03AK13, R03AL02, R03AC04, R03AC05, R03AC06, R03AC07, R03AC08, R03AC09, R03AC10, R03AC11, R03AC12, R03AC13, R03AC14, R03AC15, R03AC16, R03AC17, R03AC18, R03AC19, R03AK05, R03AK06, R03AK07, R03AK08, R03AK09, R03AK10, R03AK11, R03AK12, R03AL01, R03AL03, R03AL04, R03AL05, R03AL06, R03AL07, R03AL08, R03AL09, R03BB R03AL R03BC R03AK04, R03AK05, R03DA, R03DC, R03CC03, R03CB03, R03DX.

Hypothyroidism was defined as use of ATC H03AA medications.

All remaining conditions were identified based on self-reported doctor’s diagnosis only as it was not possible to identify medications which were specific to these conditions.

Cardiovascular disease (CVD) was defined as presence of angina, heart attack, chronic heart failure, stroke or transient ischaemic attack (TIA).

## Development of Disease Trajectories

A latent Markov model with a multivariate categorical response was implemented to develop longitudinal disease trajectories. This method has the advantage that it allows membership of latent classes to change at each wave of data collection and therefore allows us to track the evolution of disease class over time. The overall model estimates three main parameters: 1. The initial probabilities, which can be interpreted as the prevalence or size of the disease classes at baseline 2. the transition probabilities, which show the probability of moving between classes over time and 3. the conditional response probabilities, which estimate the probability of each disease being present, conditional on class membership. These conditional response probabilities can be used to aid in the interpretation of the disease classes. The observed/expected ratio was calculated by dividing the proportion of individuals with a given disease within a class by the proportion of individuals with the disease in the overall cohort. Disease class names were based on observed/expected ratio of ≥2. A covariate distinguishing the three biomarker signatures as well as the covariates described in the Variables section were added to the measurement model and allowed to affect the initial probabilities. This allowed the association between the biomarker signatures and the initial probability of belonging to a given disease class to be assessed [42].

**Supplementary References**

1. Briggs, R., et al., *Validation of the 8-item Centre for Epidemiological Studies Depression Scale in a cohort of community-dwelling older people: data from The Irish Longitudinal Study on Ageing (TILDA).* Eur Geriatr Med, 2018. **9**(1): p. 121-126.

2. O’Halloran, A.M., R.A. Kenny, and B.L. King-Kallimanis, *The latent factors of depression from the short forms of the CES-D are consistent, reliable and valid in community-living older adults.* European Geriatric Medicine, 2014. **5**(2): p. 97-102.

3. De Looze, C., et al., *Physical Function, An Adjunct to Brain Health Score for Phenotyping Cognitive Function Trajectories in Older Age: Findings From The Irish Longitudinal Study on Ageing (TILDA).* J Gerontol A Biol Sci Med Sci, 2022. **77**(8): p. 1593-1602.

# Appendix 3

**Summary of 18 biomarkers measured in TILDA and HRS – function, body system and associated outcomes**

| **No.** | **Biomarker** | **Function** | **System** | **Outcome** | **Good for Optimal Health** | **Clinical Rational** |
| --- | --- | --- | --- | --- | --- | --- |
| 1 | Systolic Blood Pressure  (SBP) | This is a measure of arterial pressure when the heart beats. Clinically high values ≥140 mmHg. | Cardiometabolic | Mortality, CVD, stroke, chronic kidney disease. | Lower | Reduced arterial strain; lower risk of stroke and heart attack. |
| 2 | Diastolic Blood Pressure  (DBP) | This is a measure of arterial pressure when the heart is at rest between beats. Clinically high values ≥90 mmHg. | Cardiometabolic | Mortality, CVD, stroke, chronic kidney disease. | Lower | Indicates healthier vascular resistance. |
| 3 | Resting Heart Rate  (RHR) | Number of palpitations made by the heart over a period of time. | Cardiometabolic | Mortality, CVD | Lower | Indicates higher cardiovascular fitness and efficiency. |
| 4 | Body Mass Index  (BMI) | A surrogate estimate of body fat based on a person’s weight (kg) divided by height (m^2^). | Cardiometabolic | Mortality, CVD, metabolic syndrome, Type 2 Diabetes | Lower (in healthy range) | Lower risk of metabolic syndrome, diabetes and heart disease |
| 5 | High density lipoprotein cholesterol  (HDL) | Metabolism.  Transports cholesterol to tissues and removes excess cholesterol in the blood and carries it back to the liver where it is converted for excretion as bile acids. Low levels promote atherosclerosis. | Cardiometabolic | Mortality, CVD, metabolic syndrome | Higher | Helps clear cholesterol from the bloodstream. |
| 6 | Low density lipoprotein cholesterol  (LDL) | Metabolism.  A primary carrier of cholesterol in blood delivering cholesterol to both peripheral (adrenal gland, gonads, muscle, and adipose tissue) and liver cells. Atherosclerosis develops when oxidized LDL-cholesterol is taken up by macrophages and deposited in plaques in the endothelial vasculature. | Cardiometabolic | Mortality, CVD, metabolic syndrome | Lower | Reduced plaque buildup (atherosclerosis) in arteries. |
| 7 | Triglycerides  (TRIG) | Metabolism.  They serve as a source of energy for the body, are stored in adipose tissue and can be broken down to release energy when needed. Triglycerides also help to insulate the body and protect vital organs. Excess levels contribute to arteriosclerosis. | Cardiometabolic | Mortality, CVD, metabolic syndrome | Lower | Reduced risk of heart disease and pancreatitis. |
| 8 | N-terminal pro B-type natriuretic peptide  (NTproBNP) | Cardiovascular.  Produced in response to stretching and damage to cardiac muscle. It has a central role in the regulation of blood pressure, blood volume, and sodium balance. | Cardiometabolic | Mortality, cardiac failure, CVD. | Lower | Marker of ventricular stretch/stress; low levels indicate healthy heart function. |
| 9 | C-reactive protein  (CRP) | Inflammation.  An acute-phase protein produced by the liver that increases following interleukin-6 secretion by macrophages and T cells. It is a marker of inflammation. | Inflammatory response | Mortality, CVD, frailty , cancer | Lower | General marker of systemic inflammation. |
| 10 | Interleukin-1 Receptor Antagonist (IL1-RA) | Inflammation.  IL-1RA is a member of the interleukin 1 cytokine family. It is secreted by various cell types including immune cells, epithelial cells, and adipocytes, and is a natural inhibitor of the pro-inflammatory effect of IL1β. It inhibits the activities of interleukin 1, alpha (IL1A) and interleukin 1, beta (IL1B), and modulates a variety of interleukin 1 related immune and inflammatory responses. | Inflammatory response | Auto-immune and inflammatory diseases | Optimal balance | Blocks pro-inflammatory action; extremely high values may signal high underlying inflammation. |
| 11 | Interleukin-6  (IL-6) | Inflammation.  Pro- (and anti-) inflammatory cytokine induced by stimulation of pattern-recognition receptors (e.g., TLRs, NLRs) and mediated by the transcription factor NFκB. | Inflammatory  response | Mortality, CVD mortality and events, Frailty, Disability, Functional decline,  Psychosocial stress. | Lower | Pro-inflammatory cytokine associated with chronic disease. |
| 12 | Interleukin-10  (IL-10) | Inflammation.  Context-dependent pleiotropic cytokine. Induced by pattern-recognition receptors (eg TLRs, NLRs) and can block NFκB proinflammatory signalling. Induced by IL-6 and induces TNFα expression. | Inflammatory  response | Autoimmune disorders, Cancers, Metabolic syndrome,  Cardiac fibrosis, Psychosocial stress. | Higher | Anti-inflammatory cytokine that suppresses chronic inflammation. |
| 13 | Tumour Necrosis Factor-alpha  (TNF-α) | Inflammation. inflammatory cytokine produced by macrophages/monocytes during acute inflammation and is responsible for a diverse range of signalling events within cells, leading to necrosis or apoptosis. | Inflammatory response | Mortality, CVD, Cancer mortality, Dementia. | Lower | Pro-inflammatory cytokine linked to chronic illness. |
| 14 | Creatinine  (CRE) | A waste by-product of muscle metabolism, used to estimate glomerular filtration of the kidney. | Renal | Mortality, Chronic kidney disease | Lower (in healthy range) | High levels indicate reduced kidney filtration capacity. |
| 15 | Cystatin C  (CYSC) | Endogenous cysteine proteinase inhibitor produced in all body fluids, used to estimate glomerular filtration of the kidney. | Renal | Mortality, Chronic kidney disease | Lower | Marker of impaired kidney function |
| 16 | Brain-derived Neurotrophic Factor (BDNF) | Cognitive function.  A neurotrophin implicated in neuronal survival/proliferation by activation of antioxidant enzymes ameliorating oxidative stress. It is a neurotransmitter regulator. | Cognition | Cognitive frailty, Cognitive impairment, AD and Depression. | Higher | Supports neuroplasticity memory and neuronal survival. |
| 17 | Leukocyte telomere length  (TL) | Cellular senescence. Telomere shortening leads to leading to accumulation of senescent cells in tissues and organs ultimately leading to loss of function over time. | Leukocyte senescence | Mortality, CVD, cancer, psychological stress | Longer (Slowing Attrition) | Shorter length linked to more favourable biological ageing; the goal is maintenance or slowing of shortening. |
| 18 | Vitamin D3 | Multifunctional, bone health, muscle health, CV health, immune function | Musculoskeletal | Mortality, frailty, malnutrition | Higher (in optimal range) | Critical for immune function, bone health and mood regulation. |

# Appendix 4


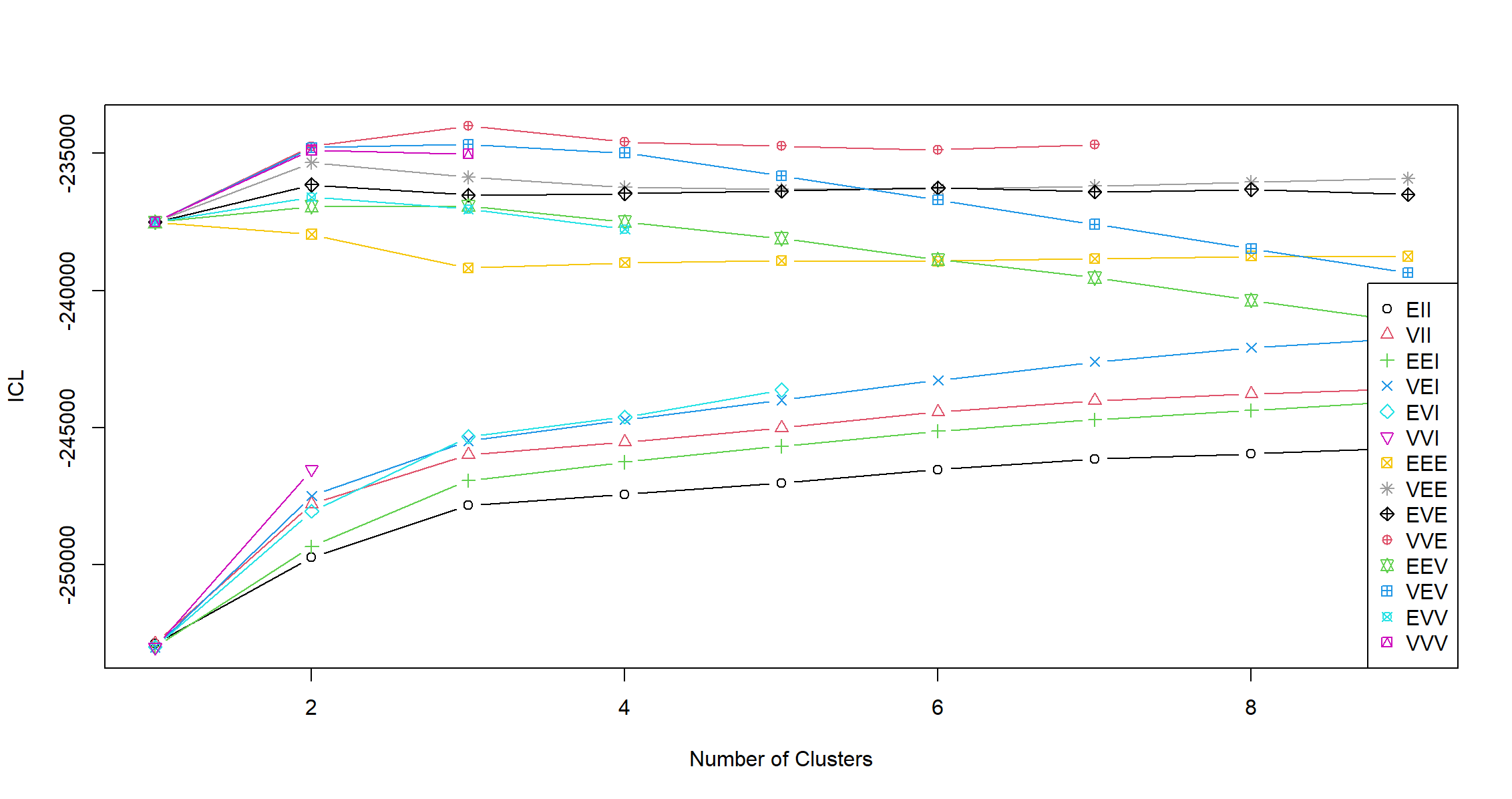


**Supplementary Figure 1**. Integrated Complete-data Likelihood (ICL) criterion for 1 to 9 clusters over 14 different constraints on the eigenvalue decomposition of the covariance matrix for TILDA bliomarker data. The 3 cluster solution using the VVE distribution structure which assumes an ellipsoidal distribution with variable volume and equal shape and orientation were chosen as this was the solution which maximised the ICL.


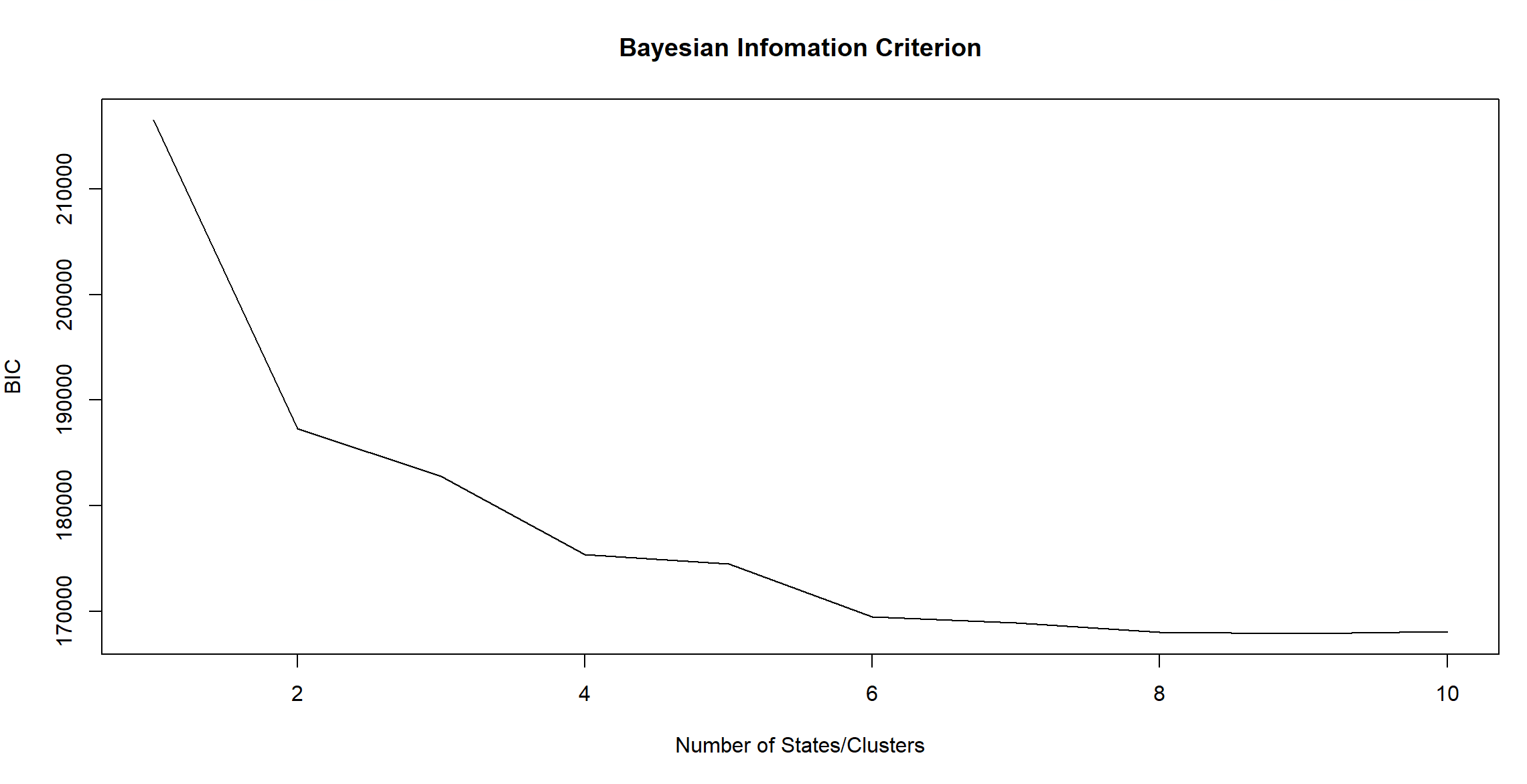


**Supplementary Figure 2.** Bayesian Information Criterion (BIC) for 1 to 10 disease classes using TILDA data. Six classes were chosen in the final model as there were only very incremental improvements in the BIC beyond this point.

**
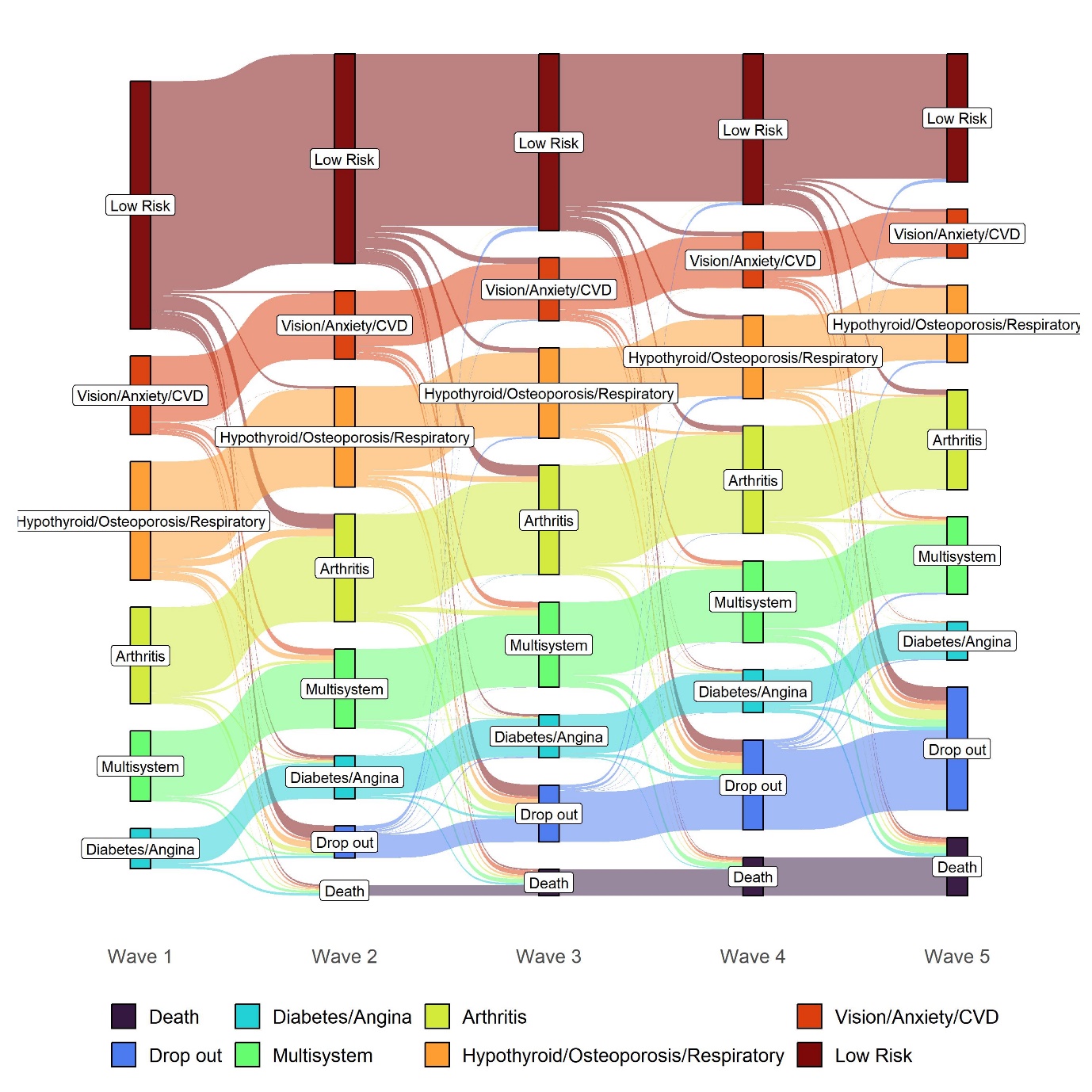
**

**Supplementary Figure 3.** Alluvial plot showing participant transitions between disease classes at each wave of data collection over 9 years in the TILDA cohort.

# Appendix 5

**Supplementary code**

#need to create same data format for bio-markers:

#This will run on the 20 markers in common between TILDA and HRS

library(readstata13)

w1biomarkers<-read.dta13("PATHTODATA")

#################################################################################################################

names<-c("zseatedRHR","zlogsystolic" ,"zlogdiastolic", "zlogFRbmi",

"zlogHDL", "zlogLDL", "zlogTRIG",

"zlogCREA", "zlogCYSC","zlogBDNF",

"zlogNTpBNP","zlogCRP","zlogIL1RA" ,"zlogIL6","zlogIL10" ,

"zlogTNFa","zlogVITD3","zLOGTELOMERE_PLATE")

#run model based clustering on the data of 4961 participants:

library(mclust)

library(mix)

#impute 100 times:

#summary(BIOM_ImputerData)

ICL_List<-list()

length(ICL_List)<-100

Best_model<-rep(NA,100)

MaxNumClust<-rep(NA,100)

library(dplyr)

set.seed(100)

for(i in 1:100){

print(c("i is",i))

set.seed(i)

BIOM_ImputerData<-imputeData(as.matrix(w1biomarkers[,names]),seed=i)

ICL <- mclustICL(BIOM_ImputerData)

ICL_List[[i]]<-ICL[,c(1:14)]

Best_model[i]<-which.max(data.frame(ICL_List[[i]]) %>% summarise_if(is.numeric, max))

MaxNumClust[i]<-which.max(data.frame(ICL_List[[i]])[,Best_model[i]])

rm(list=ls()[! ls() %in% c("w1biomarkers","names","ICL_List","Best_model","MaxNumClust")])

}

plot(ICL)

#to get the average over all elements of list:

Avg_Clust_Soln<-Reduce("+", ICL_List) / length(ICL_List)

Avg_Clust_Soln<-Reduce("+", lapply(ICL_List, function(x) replace(x, is.na(x), 0))) / Reduce("+", lapply(ICL_List, Negate(is.na)))

#then plot the results and choose the number of clusters

#reproduce this plot:

plot(Avg_Clust_Soln[,1],ylim=c(min(Avg_Clust_Soln,na.rm=TRUE),

max(Avg_Clust_Soln,na.rm=TRUE)),col=1,pch=1,type="b",xlab="Number of Clusters",ylab="ICL")

for(i in 2:ncol(Avg_Clust_Soln)){

lines(Avg_Clust_Soln[,i],ylim=c(min(Avg_Clust_Soln,na.rm=TRUE),max(Avg_Clust_Soln,na.rm=TRUE)),col=i,pch=i,type="b")

}

legend("bottomright",colnames(Avg_Clust_Soln),col=c(1:ncol(Avg_Clust_Soln)),pch=c(1:ncol(Avg_Clust_Soln)))

set.seed(10)

bio3_Classification<-matrix(rep(NA,nrow(w1biomarkers)*100),nrow=nrow(w1biomarkers),ncol=100)

bio3_MeanMat<-list()

length(bio3_MeanMat)<-100

bio4_all<-list()

length(bio4_all)<-100

for(i in 1:100){

set.seed(i)

BIOM_ImputerData<-imputeData(as.matrix(w1biomarkers[,names]),seed=i)

bio4_all[[i]]<-Mclust(BIOM_ImputerData,G=3,modelNames = "VVE")

bio4Out<-summary(bio4_all[[i]],parameters=TRUE)

bio3_MeanMat[[i]]<-bio4Out$mean

bio3_Classification[,i]<-bio4Out$classification

rm(BIOM_ImputerData,bio4Out)

}

#need to now match the labels across iterations as label 1 in iteration 1 isn't necessarily called 1 in iteration2 etc

#R gives the new labels matched to iteration 1

#map all labels to iteration 1:

bio3_MeanMat_Remaped<-list()

length(bio3_MeanMat_Remaped)<-100

bio3_ClassRemapped<-matrix(rep(NA,nrow(w1biomarkers)*100),nrow=nrow(w1biomarkers),ncol=100)

bio3_ClassRemapped[,1]<-bio3_Classification[,1]

bio3_MeanMat_Remaped[[1]]<-bio3_MeanMat[[1]]

library(Thresher)

set.seed(1)

for(i in 1:100){

#R remaps to the first iteration

R <- remap(bio3_Classification[,1], bio3_Classification[,i])

bio3_ClassRemapped[,i]<-R

#now reorder the columns of meanmat

#take col 1 as the ground truth and match everything to that.....

#relabel the clusters to match iteraton 1 and reorder the mean matrices so they have the same order:

tab <- table(bio3_Classification[,1], bio3_Classification[,i])

MLabs<-matchLabels(tab)

bio3_MeanMat_Remaped[[i]]<-bio3_MeanMat[[i]][,as.numeric(colnames(MLabs))[order(as.numeric(rownames(MLabs)))]]

}

#get average membership:

Avg_Clust4<-apply(with(stack(data.frame(t(bio3_ClassRemapped))), table(ind, values)),1,function(x) which.max(x))

table(Avg_Clust4)

round(prop.table(table(Avg_Clust4)),3)*100

#####################################################################################

#now draw a heat map of the cluster means, over the 100 iterations:

meanmat4<-Reduce("+", bio3_MeanMat_Remaped) / length(bio3_MeanMat_Remaped)

colnames(meanmat4)<-c("Group 1: Low Risk","Group 2: High Risk","Group 3: Medium Risk")

#########################################################################################

library(ggplot2)

library(reshape2)

library(RColorBrewer)

library("lattice")

coul <- colorRampPalette(brewer.pal(11, "PuOr"))(250)

brk<-do.breaks(c(-2.2,2.2), 250)

levelplot(value ~ Var2*Var1, data=melt(meanmat4[,]) ,scales=list(x=list(rot=45)) ,xlab="",ylab="",at = brk,

colorkey = list(col = coul,

at = brk),

main="",col.regions = coul)

library(survival)

library(survminer)

library(dplyr)

surv_object <- Surv(time = AllMortality$wave1age+AllMortality$timew1interviewtodeath , event = AllMortality$status)

plot(survfit(surv_object~ AllMortality$Mclust4),col=AllMortality$Mclust4)

surv_object2 <- Surv(time = AllMortality$timew1interviewtodeath , event = AllMortality$status)

plot(survfit(surv_object2~ AllMortality$Mclust4),col=AllMortality$Mclust4)

AllMortality$Mclust4<-as.factor(AllMortality$Mclust4)

AllMortality$Mclust4<-relevel(AllMortality$Mclust4,"1")

fit <- coxph(surv_object2 ~ bloodage+as.factor(sex.x)+as.factor(edu3)+BEHsmoker+MDantihypertensives2+MDlipmod+FRbmi+Mclust4, data = AllMortality)

summary(fit)

anova(fit)

test.ph <- cox.zph(fit)

test.ph

ggcoxzph(test.ph)

summary(fit)$concordance

#now test accuracy (training for TILDA and Test for HRS):

library(dynpred)

cindex <- cindex(surv_object2 ~ bloodage+as.factor(sex.x)+as.factor(edu3)+BEHsmoker+MDantihypertensives2+MDlipmod+FRbmi+Mclust4, data = AllMortality)

cindex

#######################################################################################################################

####identify longitudinal disease trajectories and see if biomarker clusters are predictive:

#######################################################################################################################

#change the cluster names for the models from now on:

w1biomarkers$Mclust4<-factor(w1biomarkers$Mclust4,levels=c(1,3,2))

levels(w1biomarkers$Mclust4)<-list("Low Risk"="1","Medium Risk"="3", "High Risk"=2)

table(w1biomarkers$Mclust4)

library(nlme)

m1 <- lme(FRtugTimeSec~age+sex+edu3+BEHsmoker+MDantihypertensives2+MDlipmod+Mclust4+Wave,random=~1|tilda_serial,data=TUGTIME_Long)

#wave is significant but interaction between wave and group isn't

anova(m1)

summary(m1)

#get the RMSE

TugTime.rmse <- sqrt(mean(m1$residuals^2))

TugTime.rmse

library(ggeffects)

Tug <- ggpredict(m1, c("Wave","Mclust4"))

Tug

Tug_plot<-plot(Tug)+labs(y="TUG Time", title="Predicted TUG Time")+theme_classic()+theme(legend.title=element_blank())

Tug_plot

##############################################################

#now do same for grip strength at waves 1 and 3

###############################################################

w1biomarkers<-merge(w1biomarkers,w1[,c("tilda_serial","GRTspeed")],by="tilda_serial")

#########################################################################################

#center age around 50

GRT_Long$age<-GRT_Long$age-50

GRT_Long$Mclust4<-factor(GRT_Long$Mclust4)

library(nlme)

#clusters not significant in predicting gait.

Gair_lme <- lme(GRTspeed~age+sex+edu3+BEHsmoker+MDantihypertensives2+MDlipmod+Mclust4*Wave,random=~1|tilda_serial,data=GRT_Long)

#get the RMSE

Gait.rmse <- sqrt(mean(Gair_lme$residuals^2))

#wave is significant but interaction between wave and group isn't

anova(Gair_lme)

summary(Gair_lme)

#now plot the differences:

library(ggeffects)

pr <- ggpredict(Gair_lme, c("Wave","Mclust4"))

pr

Gait_plot<-plot(pr)+labs(y="Usual Gait Speed", title="Predicted Gait Speed")+theme(legend.title=element_blank())+theme_classic()

Gait_plot

##############################################################

#now do same for grip strength at waves 1 and 3

###############################################################

#########################################################################################

#center age around 50

GS_Long$Wave<-as.factor(GS_Long$Wave)

library(nlme)

#clusters not significant in predicting tug.

GS_lme <- lme(FRgripstrengthmean~age+sex+edu3+BEHsmoker+MDantihypertensives2+MDlipmod+Mclust4*Wave,random=~1|tilda_serial,data=GS_Long)

#wave is significant but interaction between wave and group isn't

anova(GS_lme)

summary(GS_lme)

#get the RMSE

GS.rmse <- sqrt(mean(GS_lme$residuals^2))

#now plot the differences:

library(ggeffects)

pr <- ggpredict(GS_lme, c("Wave","Mclust4"))

pr

GStrength_plot<-plot(pr)+labs(y="Grip Strength", title="Predicted Grip Strength")+theme_classic()+theme(legend.title=element_blank())

GStrength_plot

#can see that wave 2 in general looks off....its far higher than other waves

#overall cluster was significant, and cluster by time was significant cluster 1 had sign. lower GS across all waves except baseline.

rm(GS_Long,TUGTIME_Long)

library(ggpubr)

ConOutcomes_Plot <- ggarrange(GStrength_plot,Gait_plot, Tug_plot,

ncol = 3, nrow = 1,common.legend = TRUE,legend="bottom")

jpeg("Fig1C_ContinuousOutcomes.jpeg", width = 10, height = 5, units = 'in', res = 300)

ConOutcomes_Plot

dev.off()

#Incident Disability:

DISABL_Incidence<-w1biomarkers[-c(which(w1biomarkers$DISany==1)),]

dim(w1biomarkers)

dim(DISABL_Incidence)

#removed 254 from baseline

#now create binary variable for DISABL indicence over next 8 years:

#first do a complete case analysis of DISABL indidence in those who were in all 5 waves:

DISABL_INCALL<-ifelse(DISABL_Incidence$DISABL2==1|DISABL_Incidence$DISABL3==1|DISABL_Incidence$DISABL4==1|DISABL_Incidence$DISABL5==1,1,0)

DISABL_Incidence$age<-DISABL_Incidence$age-50

DISABLINC<-glm(DISABL_INCALL~age+sex+edu3+BEHsmoker+MDantihypertensives2+MDlipmod+Mclust4,family=binomial,data=DISABL_Incidence)

DISABLINC2<-glm(DISABL_INCALL~age+sex+edu3+BEHsmoker+MDantihypertensives2+MDlipmod,family=binomial,data=DISABL_Incidence)

summary(DISABLINC)

anova(DISABLINC,DISABLINC2,test="LRT")

print(tidy(DISABLINC, conf.int = TRUE, conf.level = 0.95, exponentiate = TRUE),n=200)

Mortality_Model<-fit4

#now get accuracy measure AUC and sensitivity and specificity:

library(pROC)

Dis_pred<-predict(DISABLINC,newdata=DISABL_Incidence,type="response")

AUC_DISabl<-roc(DISABL_INCALL,Dis_pred)

ci.auc(AUC_DISabl)

library(caret)

#the prevalence of disability os 0.1 so I set the threshold equal to this for predicting disability:

confusionMatrix(as.factor(ifelse(Dis_pred>0.1,1,0)),as.factor(DISABL_INCALL))

##############################################################################################################

#Now look at frailty!

##############################################################################################################

frailty_dat<-read.dta13("PATHFILE")

w1biomarkers<-merge(w1biomarkers,frailty_dat[,c("tilda_serial","FRgroup_w1")] ,by="tilda_serial",all.x=TRUE)

Frailty_Long<-w1biomarkers[,c("tilda_serial","FRgroup_w1","Mclust4",

"BEHsmoker","MDantihypertensives2","MDlipmod","edu3","sex", "age")]

FRAIL_INCALL<-ifelse(FRAIL_Incidence$FRgroup_w2>1|FRAIL_Incidence$FRgroup_w3>1|FRAIL_Incidence$FRgroup_w4>1|FRAIL_Incidence$FRgroup_w5>1,1,0)

FRAIL_Incidence$age<-FRAIL_Incidence$age-50

table(FRAIL_INCALL)

FRAILINC<-glm(FRAIL_INCALL~age+sex+edu3+BEHsmoker+MDantihypertensives2+MDlipmod+Mclust4,family=binomial,data=FRAIL_Incidence)

FRAILINC2<-glm(FRAIL_INCALL~age+sex+edu3+BEHsmoker+MDantihypertensives2+MDlipmod,family=binomial,data=FRAIL_Incidence)

summary(FRAILINC)

anova(FRAILINC,FRAILINC2,test="LRT")

print(tidy(FRAILINC, conf.int = TRUE, conf.level = 0.95, exponentiate = TRUE),n=200)

library(pROC)

FRAIL_pred<-predict(FRAILINC,newdata=FRAIL_Incidence,type="response")

AUC_FRAIL<-roc(as.factor(FRAIL_INCALL),FRAIL_pred)

ci.auc(AUC_FRAIL)

library(caret)

confusionMatrix(as.factor(ifelse(FRAIL_pred>0.22,1,0)),as.factor(FRAIL_INCALL))

rm(FRAIL_Incidence)

#############################################################

# Multimorbidity #

#############################################################

#look at incident MM?

MM_Incidence<-w1biomarkers[-c(which(w1biomarkers$MM_Score>=2)),]

dim(w1biomarkers)

dim(MM_Incidence)

#removed 1582 from baseline

#groups 1 and 4 have higher odds of incident MM:

MMINC<-glm(MM_INCALL~age+sex+edu3+BEHsmoker+MDantihypertensives2+MDlipmod+Mclust4,family=binomial,data=MM_Incidence)

MMINC2<-glm(MM_INCALL~age+sex+edu3+BEHsmoker+MDantihypertensives2+MDlipmod,family=binomial,data=MM_Incidence)

anova(MMINC,MMINC2,test="LRT")

print(tidy(MMINC, conf.int = TRUE, conf.level = 0.95, exponentiate = TRUE),n=200)

#now get accuracy

library(pROC)

MM_pred<-predict(MMINC,newdata=MM_Incidence,type="response")

AUC_MM<-roc(as.factor(MM_INCALL),MM_pred)

ci.auc(AUC_MM)

library(caret)

confusionMatrix(as.factor(ifelse(MM_pred>0.5,1,0)),positive ="1",as.factor(MM_INCALL))

#############################################################

# Incident CVD #

#############################################################

CVD_Incidence<-w1biomarkers[-c(which(w1biomarkers$PREV_W1_angina==1|w1biomarkers$PREV_W1_heartattack==1|w1biomarkers$PREV_W1_chf==1|w1biomarkers$PREV_W1_stroke==1|w1biomarkers$PREV_W1_tia==1)),]

#now create binary variable for CVD indicence over next 8 years:

CVDINC<-glm(CVD_INCALL~age+sex+edu3+BEHsmoker+MDantihypertensives2+MDlipmod+Mclust4,family=binomial,data=CVD_Incidence)

summary(CVDINC)

anova(CVDINC,test="LRT")

#what about controlling for use ofr antihypertensives at baseline?

print(tidy(CVDINC, conf.int = TRUE, conf.level = 0.95, exponentiate = TRUE),n=200)

#now get accuracy measure AUC and sensitivity and specificity:

library(pROC)

CVD_pred<-predict(CVDINC,newdata=CVD_Incidence,type="response")

AUC_CVD<-roc(as.factor(CVD_Incidence$CVD_INCALL),CVD_pred)

ci.auc(AUC_CVD)

library(caret)

#the prevalence of disability os 0.5 so I set the threshold equal to this for predicting disability:

confusionMatrix(as.factor(ifelse(CVD_pred>0.5,1,0)),as.factor(CVD_Incidence$CVD_INCALL),positive="1")

rm(CVD_Incidence)

#############################################################

# Incident Diabetes #

#############################################################

Diabetes_Incidence<-w1biomarkers[-c(which(w1biomarkers$PREV_W1_diabetes==1)),]

DiabetesINC<-glm(Diabetes_INCALL~age+sex+edu3+BEHsmoker+MDantihypertensives2+MDlipmod+Mclust4,

family=binomial,model=FALSE, y=FALSE,data=Diabetes_Incidence)

summary(DiabetesINC)

DiabetesIN2<-glm(Diabetes_INCALL~age+sex+edu3+BEHsmoker+MDantihypertensives2+MDlipmod,family=binomial,data=Diabetes_Incidence)

anova(DiabetesINC,DiabetesIN2,test="LRT")

print(tidy(DiabetesINC, conf.int = TRUE, conf.level = 0.95, exponentiate = TRUE),n=200)

#now get accuracy measure AUC and sensitivity and specificity:

library(pROC)

Diabetes_pred<-predict(DiabetesINC,newdata=Diabetes_Incidence,type="response")

AUC_Diabetes<-roc(as.factor(Diabetes_INCALL),Diabetes_pred)

ci.auc(AUC_Diabetes)

library(caret)

#the prevalence of disability os 0.5 so I set the threshold equal to this for predicting disability:

confusionMatrix(as.factor(ifelse(CVD_pred>0.05,1,0)),as.factor(CVD_INCALL))

save(Mortality_Model,DISABLINC,FRAILINC,MMINC,CVDINC,DiabetesINC,file="MclustHRS_PREDICTION_MODELS.Rdata")

rm(Diabetes_Incidence)

#############################################################

library(LMest)

data("RLMSlong")

dim(RLMSlong)

head(RLMSlong)

library(readstata13)

####################################################################################

#####################################################################################

#run summary of data by year

library(LMest)

set.seed(100)

dt <- lmestData(data = temp_data, id = "tilda_serial", time="time",

responsesFormula= PREV_angina

+PREV_armd+PREV_arthritis+PREV_Thyroid+PREV_cancer+PREV_cataract+CHF_HA+COG_Imp+Pain+

+PREV_diabetes+STROKE_TIA+#PREV_highchol+PREV_hbp+

PREV_lungdiseaseALL+PREV_HEARTRHYTHM+PREV_depression+PREV_anxiety

+PREV_glaucoma+PREV_osteoporosis~NULL)

summary(dt)

mmtraj_bic<-mmtraj_aic<-mmtraj_lk<-rep(NA,10)

set.seed(100)

for(i in 1:10){

mod <- lmest(responsesFormula = PREV_angina

+PREV_armd+PREV_arthritis+PREV_Thyroid+PREV_cancer+PREV_cataract+CHF_HA+COG_Imp+Pain+

+PREV_diabetes+STROKE_TIA+#PREV_highchol+PREV_hbp+

PREV_lungdiseaseALL+PREV_HEARTRHYTHM+PREV_depression

+PREV_glaucoma+PREV_osteoporosis+PREV_anxiety~ NULL,

index = c("tilda_serial","time"),

data = temp_data, k = i)

mmtraj_bic[i]<-mod$bic

mmtraj_aic[i]<-mod$aic

mmtraj_lk[i]<-mod$lk

}

print(mod)

plot(1:10,mmtraj_bic,type="l",xlab="Number of States/Clusters",ylab="BIC",main="Bayesian Infomation Criterion")

set.seed(10)

#6 clusters and covariates in the latent model

mod_latentcov7_initial <- lmest(responsesFormula =PREV_angina

+PREV_armd+PREV_arthritis+PREV_Thyroid+PREV_cancer+PREV_cataract+CHF_HA+COG_Imp+Pain+

+PREV_diabetes+STROKE_TIA+PREV_highchol+PREV_hbp+

PREV_lungdiseaseALL+PREV_HEARTRHYTHM+PREV_depression

+PREV_glaucoma+PREV_osteoporosis + PREV_anxiety~NULL,

latentFormula = ~age_corr + sex + edu3+

BEHsmoker +MDlipmod +MDantihypertensives2|NULL,paramLatent = "multilogit",

index = c("tilda_serial","time"),

data = temp_data, k = 6,output=TRUE,out_se=TRUE,seed = 123)

summary(mod_latentcov7_initial)

#covariates affecting the initial states

mod_latentcov7_initial$Be

#intercepts for transition probs

mod_latentcov7_initial$Ga

round(table(mod_latentcov7_initial$Ul[,5])/nrow(mod_latentcov7_initial$Ul),3)*100

mod_latentcov7_initial$PI

plot(mod_latentcov7_initial, what = "transitions")

plot(mod_latentcov7_initial, what="marginal")

#get p-values

semodc<-se(mod_latentcov7_initial)

TabBe <-cbind(mod_latentcov7_initial$Be, semodc$seBe, mod_latentcov7_initial$Be/semodc$seBe)

colnames(TabBe) <- c("estGa(2)","estGa(3)","estGa(4)","estGa(5)","estGa(6)",

"s.e.Ga(2)","s.e.Ga(3)","s.e.Ga(4)","s.e.Ga(5)","s.e.Ga(6)",

"t-test(2)","t-test(3)","t-test(4)","t-test(5)","t-test(6)")

round(TabBe,3)

pnorm(-abs(mod_latentcov7_initial$Be)/semodc$seBe)*1.96

#prevalences

piv1 <- round(colMeans(mod_latentcov7_initial$Piv), 4)

#transition matrix

PI1 <- round(apply(mod_latentcov7_initial$PI[, , , 2:5], c(1, 2), mean), 4)

###################################################################

#prevalence of each state:

table(mod_latentcov7_initial$Ul[,1])/nrow(mod_latentcov7_initial$Ul)

deccoding<-as.data.frame(mod_latentcov7_initial$Ul)

deccoding$tilda_serial<-temp_data1$tilda_serial

deccoding<-merge(deccoding,AllMortality[,c("Deceased_wave","Deceased","tilda_serial","in_capi_W2","in_capi_W3","in_capi_W4","in_capi_W5")],by="tilda_serial")

library(ggsankey)

library(ggplot2)

library(dplyr)

colnames(deccoding)[2:6]<-c("Wave 1","Wave 2","Wave 3","Wave 4","Wave 5")

df <- deccoding[,2:6] %>%

make_long(`Wave 1`, `Wave 2`, `Wave 3`, `Wave 4`,`Wave 5`)

#change cluster names:

df_Names<-df

df_Names$node[which(df_Names$node==1)]<-"Drop out"

df_Names$next_node[which(df_Names$next_node==1)]<-"Drop out"

#dead

df_Names$node[which(df_Names$node==2)]<-"Death"

df_Names$next_node[which(df_Names$next_node==2)]<-"Death"

#Multisystem

df_Names$node[which(df_Names$node==3)]<-"Diabetes/Angina"

df_Names$next_node[which(df_Names$next_node==3)]<-"Diabetes/Angina"

#4CVD/Vision/Arthritis

df_Names$node[which(df_Names$node==4)]<-"Multisystem"

df_Names$next_node[which(df_Names$next_node==4)]<-"Multisystem"

#4Respiratory

df_Names$node[which(df_Names$node==5)]<-"Arthritis"

df_Names$next_node[which(df_Names$next_node==5)]<-"Arthritis"

#Arthritis

df_Names$node[which(df_Names$node==6)]<-"Hypothyroid/Osteoporosis/Respiratory"

df_Names$next_node[which(df_Names$next_node==6)]<-"Hypothyroid/Osteoporosis/Respiratory"

#Osteoporosis

df_Names$node[which(df_Names$node==7)]<-"Vision/Anxiety/CVD"

df_Names$next_node[which(df_Names$next_node==7)]<-"Vision/Anxiety/CVD"

#CVD/Vision

df_Names$node[which(df_Names$node==8)]<-"Low Risk"

df_Names$next_node[which(df_Names$next_node==8)]<-"Low Risk"

######################################################################

deccoding<-merge(deccoding,w1biomarkers[,c("tilda_serial","age","sex","edu3",

"BEHsmoker" ,"MDlipmod" ,"MDantihypertensives2","Mclust4")],by="tilda_serial")

library(nnet)

w5_diseaseClust<-summary(multinom(deccoding$W5_DiseaseClust~age+sex+edu3+BEHsmoker+MDlipmod+MDantihypertensives2+Mclust4+W1_DiseaseClust,data=deccoding))

surv_object3 <- Surv(time = All_mortDisease$timew1interviewtodeath , event = All_mortDisease$status)

prop.table(table(All_mortDisease$status,All_mortDisease$DiseaseStates),margin=2)

fit_DiseaseStates <- coxph(surv_object3 ~ bloodage+as.factor(sex.x)+as.factor(edu3)+BEHsmoker+MDantihypertensives2+MDlipmod+FRbmi+DiseaseStates, data = All_mortDisease)

summary(fit_DiseaseStates)

anova(fit_DiseaseStates)

test.ph <- cox.zph(fit_DiseaseStates)

test.ph

ggcoxzph(test.ph)

summary(fit_DiseaseStates)$concordance
